# Longitudinal angiographic characterization of the efficacy of combined cerebral revascularization using minimally invasive encephalodurosynangiosis in patients with moyamoya angiopathy

**DOI:** 10.1007/s10143-022-01862-9

**Published:** 2022-09-26

**Authors:** K. Lucia, G. Acker, F. Mrosk, D. Beyaztas, Peter Vajkoczy

**Affiliations:** 1 Department of Neurosurgery, Charité – Universitätsmedizin Berlin corporate member of Freie Universität Berlin and Humboldt-Universität zu Berlin, Charitéplatz 1, 10117 Berlin, Germany; 2 Berlin Institute of Health at Charité – Universitätsmedizin Berlin, Clinician Scientist Program, Charitéplatz 1, 10117 Berlin, Germany

**Keywords:** Moyamoya, Encephalodurosynangiosis, Bypass, Angiogenesis, Angiography

## Abstract

Moyamoya angiopathy (MMA) can be treated using direct, indirect, or combined revascularization procedures. We perform combined revascularization using the STA-MCA bypass and minimally invasive encephalodurosynangiosis (MIS-EDS). Due to lack of systematic analyses to date it remains unclear whether and to which extent this limited EDS serves as a growth source for extracerebral blood vessels into the brain. The objective of the current study is to characterize the extent of angiographic filling of MIS-EDS and STA-MCA bypass development over time and to determine possible predictors of EDS development in adult MMA patients. Single-center retrospective analysis of 81 MMA patients (139 hemispheres) treated with a MIS-EDS and STA-MCA bypass was performed. Angiographic images and clinical/operative data were reviewed and scored. Uni-/ and multivariate Cox regression analyses identified preoperative predictors of good EDS vascularization. At 3–6 months after surgery EDS showed moderate and high angiographic filling in 40% and 5% of hemispheres, respectively. After 12 months moderate and high filling was found in 57% and 4% of hemispheres, respectively. STA-MCA bypass filling was moderate in 47% and high in 7% of hemispheres at 3–6 months and 45% moderate and 9% high after 12 months. High STA-MCA bypass filling on angiography was a negative predictor of EDS development. MIS-EDS is a simple technique and serves as a source of vessel growth into the brain. EDS development lags behind that of STA-MCA bypass and can be recommended as an additive revascularization source when combined with a direct bypass.

## Introduction

Moyamoya angiopathy (MMA) is a rare stenoocclusive cerebrovascular disease affecting the basal cerebral arteries which leads to the formation of fragile net-like vessels [1]. As such, patients with MMA can present with intracranial hemorrhage from abnormal vessels as well as ischemic events [2, 3]. Surgical revascularization aiming at restoring perfusion and preventing future hemorrhage and ischemia is widely accepted as the primary treatment for MMA [4–6]. Revascularization can be achieved by both direct (anastomosis performed between branches of the internal and external carotid arteries) and indirect techniques which rely on neoangeogenesis between the cortical surface and a pedicled graft [7]. Common indirect revascularization techniques involve collaterals from the middle meningeal artery via the dura mater (encephalodurosynangiosis, EDS), the temporalis muscle (encephalomyosynangiosis, EMS), or further variants such as encephalao-duro-arterio-synangiosis (EDAS) and encephalo-duro-arterio-myo-synangiosis (EDAMS) [8].

Studies examining the efficacy of combined revascularization procedures have provided evidence of improved angiographic revascularization when EMS [9], EDAMS [10], and EDAS [11, 12] are used in combination with STA-MCA direct bypass. Radiological studies have also shown increased cortical perfusion in patients treated with indirect revascularization techniques such as EDAS [13]. To date, the use of EDS as a further indirect revascularization technique in combination with STA-MCA direct bypass has not been angiographically characterized as to whether or not vessel growth occurs, possibly due to the simplicity of the technique as well as the small surface contact between dura and brain surface. In our institution, minimally invasive EDS (MIS-EDS) is performed via a mini-craniotomy which is significantly less invasive than other indirect techniques requiring greater exposure. We therefore aimed to characterize the angiographic bypass development of both indirect MIS-EDS and direct STA-MCA bypass over time as well as possible predictors of EDS development.

## Methods

### Study design

We retrospectively identified all moyamoya patients who were treated using a MIS-EDS as an indirect bypass strategy combined with an STA-MCA direct bypass on at least one hemisphere in our department between January 2007 and December 2019. All patients with unilateral moyamoya and moyamoya syndrome using the ICD code 167.5 (moyamoya) were included. Moyamoya angiopathy is used as to describe all forms of the disease, including moyamoya disease (MMD), moyamoya syndrome (MMS), and unilateral moyamoya disease or syndrome (uMMD/uMMS). As such, moyamoya disease and unilateral moyamoya disease were defined based on the guidelines established by the Research Committee for moyamoya disease and patients with possible associated diseases were classified as moyamoya syndrome [14].

### Variables

Electronic medical records were used to gather data on patient demographics, time of surgery, follow-up time, angiographic follow-up, history of complications, and MRI and hemodynamic diagnostics (obtained either by xenon-computerized tomography or acetazolamide-stimulated single-photon emission computed tomography).

Severity of the MMA was classified using the Suzuki and Berlin Gradings [1, 9]. Preoperative CVRC was included in the calculation of the Berlin Grading. The qualitative assessment of the CVRC was performed as described before [15]. Briefly, CVRC was assessed qualitatively by visual interpretation with a standardized side-by-side display of anatomically normalized resting and acetazolamide images together with the rCVRC map. Impaired cerebrovascular reserve capacity (CVRC) was defined as a missing signal increase after acetazolamide stimulation. If stimulation with acetazolamide leaded to an increase of the signal, CVRC was considered sustained. In 18 patients preoperative CVRC data was not available.

### Angiographic evaluation

**Fig. 1:**
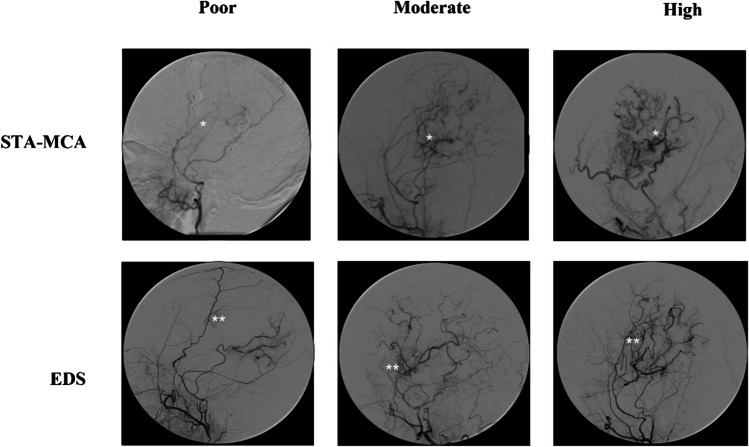
Qualitative angiographic grading of EDS and STA bypass development. Top row, single white asterisk: examples of STA-MCA bypass grading with grade 1 as “poor” (no filling of the MCA territory), grade 2 “moderate” (antegrade filing of one or two MCA branches) or grade 3 “high” (complete filling of the MCA territory). Bottom row, double white asterisk: EDS development via collaterals from the middle meningeal artery was classified either as “poor” (no filling), moderate (opacification of cortical vessels), or “high” (filling of the MCA territory)

Qualitative angiographic analysis of direct bypass development was performed as previously described by Czabanka et al. [9] with the exception that we implemented a three-grade classification instead of four in which the Czabanka grades 1 and 2 were combined to better allow for uniform comparison of diverse angiographic image sets. Accordingly, angiographic filling of STA-MCA bypasses were classified either as “poor” (no filling of the MCA territory), “moderate” (antegrade filing of one or two MCA branches), or “high” (complete filling of the MCA territory) [9]. Angiographic filling resulting from EDS was classified in adaptation to previously described methods either as “poor” (no filling), moderate (opacification of cortical vessels), or “high” (any filling of the MCA territory) [12] (Fig. 1). EDS development is therefore understood as the state of angiogenesis which has been reached at the point of angiographic analysis. In the results, EDS development will be referred to as EDS/bypass filling as it is also used for the direct bypass in order to ease comparison between both entities.

**Fig. 2:**
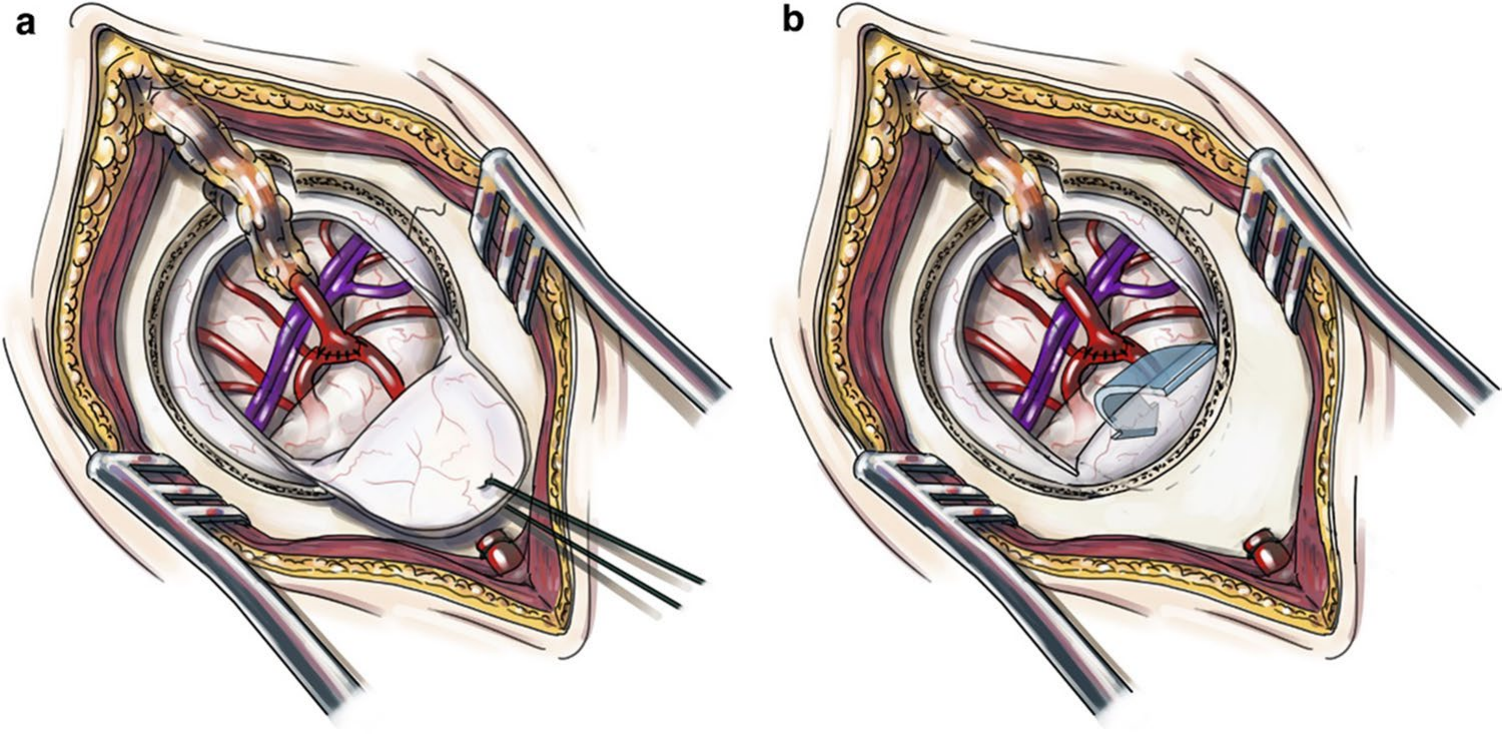
Illustration of the MIS-EDS technique. For combined revascularization with EDS, we use a minimally invasive strategy via a small craniotomy of approximately 3 cm diameter which is placed according to a standardized template [17]. **A** Following placement of the STA-MCA bypass; **B** the dural flap is inverted and laid on top of the cortex and further closure is performed as with any other craniotomy assuring the STA exits through the burr hole

In our department, the MIS-EDS was performed without changing the exposure strategy for direct revascularization perfomed via a mini-craniotomy of 3 cm in diameter [16]. At the end of the direct revascularization procedure, the dural flap was inverted and pushed beneath the bone (Fig. 2).

### Statistical analysis

Quantitative values are presented as median values with range. Univariate Cox regression analysis was performed among all cases of surgically treated MMA using “high” and “moderate” bypass filling as dependent variables. Multivariate analysis was performed on those variables which reached a *p*-value of < 0.150. All analyses were performed independently for time periods of 3–6 months, 6–12 months, 12–24 months, and > 24 months following initial revascularization. *P-*values of less than 0.05 were considered statistically significant. All analyses were performed in SPSS (version 24; IBM Corp.).

## Results

### Patient characteristics

We analyzed a total of 139 surgically treated hemispheres in 81 MMA patients. All patients were operated in a single institution between 2007 and 2019 by the senior author. Of these patients, 65 (80%) had moyamoya disease (MMD), 4 (5%) had unilateral MMD (uMMD), and 12 (15%) had moyamoya syndrome. The median age at first surgical intervention was 44 years old (range: 6–65 years old and 4 (5%) patients were under 18 years of age. Regarding gender distribution, 58 (72%) of patients were female and 28% were male. The majority of our cohort was of Caucasian background (92%), 6% were of Asian, and 2% were of Middle Eastern descent (Table 1).

**Table 1:** Patient demographics and disease characteristics. Age is reported as median and range. All other parameters as absolute number of patients or hemispheres with percent of total in parentheses

Parameter	*N* (%)
Age	44 (6–65)
Gender	
Male	23 (28%)
Female	58 (72%)
Diagnosis	
MMD	65 (80%)
MMS	12 (15%)
uMMD	4 (5%)
Onset	
Ischemic	72 (89%)
Hemorrhagic	9 (11%)
Suzuki grade	
1	14 (10%)
2	50 (36%)
3	49 (35%)
4	12 (9%)
5	12 (9%)
6	2 (1%)
Berlin grade	
1	35 (25%)
2	83 (60%)
3	21 (15%)
CVRC	
Sustained	19 (18%)
Impaired	84 (82%)
Follow-up	
Total	1.6 years (2.5 months to 6.7 years)
Complications	
Wound healing disorder	2
New Ischemia	2

The majority of our cohort (89%) presented with ischemic events leading to diagnosis of MMA with only 9 patients (11%) suffering from hemorrhagic lesions. Berlin grading of all hemispheres revealed 35 (25%) grade 1, 83 (60%) grade 2, 21 (15%) grade 3. Suzuki classification showed 14 stage 1 (10%), 50 stage 2 (36%), 49 stage 3 (35%), 12 stage 4 (9%), 12 stage 5 (9%), 2 stage 6 (1%) hemispheres. The preoperative CVRC measurement was missing in 18 patients. In the remaining 103 hemispheres 19 (18%) showed sustained function with 82% having impaired CVRC.

### Angiographic evaluation

The median follow-up period for all patients was 1.6 years (range 2.5 months to 6.7 years). For the time points, 3–6 months postoperative data was available from all 139 hemispheres, at 6–12 months from 78 hemispheres (56%), at 12–24 months for 42 hemispheres (30%), and time points beyond 24 months from 14 hemispheres (10%).

**Fig. 3:**
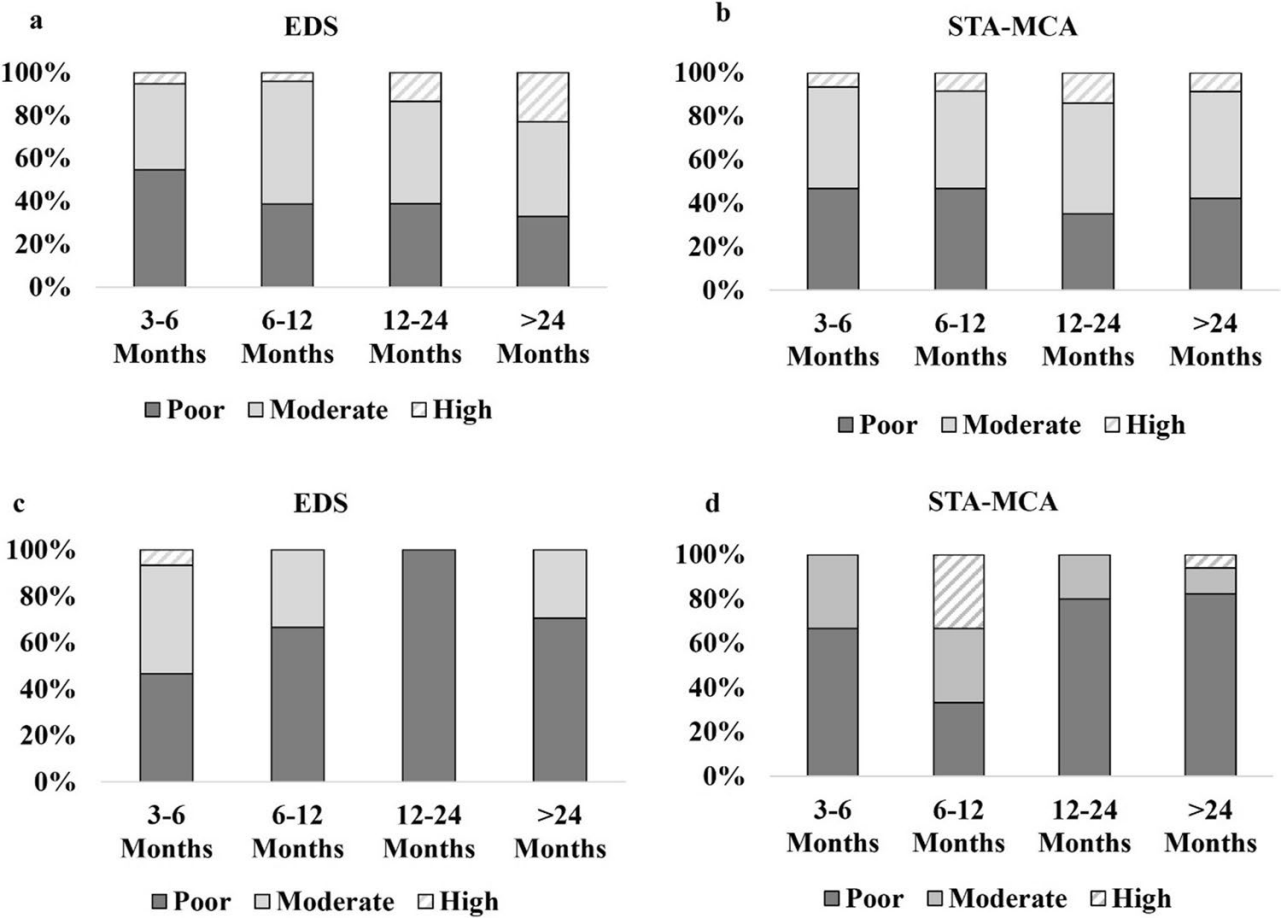
Longitudinal bypass development. **a** EDS and **b** STA-MCA bypass development in the overall cohort at the time points 3–6 months (139 hemispheres), 6–12 months (78 hemispheres), 12–24 months (42 hemispheres), and > 24 months (14 hemispheres) following surgery. Percentages represent the number of hemispheres being scored as grade 1 (poor), 2 (moderate), or 3 (high) as a total of all hemispheres undergoing analysis for that particular time period. **c** EDS and **d** STA-MCA bypass development in the subgroup of 14 hemispheres at the time points 3–6 months, 6–12 months, 12–24 months, and > 24 months following surgery. Percentages represent the number of hemispheres being scored as grade 1 (poor), 2 (moderate), or 3 (high) as a total of all hemispheres undergoing analysis for that particular time period

We first performed analysis on the overall cohort of 139 hemispheres (Fig. 3a, b) followed by a subgroup consisting of the 14 hemispheres in which data was available from all time points beyond 24 months (Fig. 3c, d).

#### EDS development and STA-MCA bypass filling within the overall patient cohort

Within the overall cohort, rates of poor bypass filling were seen more frequently (55%) in EDS than STA-MCA (47%) at 3–6 months following combined revascularization. Accordingly, higher rates of moderate bypass filling were 46% of STA-MCA compared to 40% in EDS. The rate of high bypass filling was similar between EDS (5%) and STA-MCA (7%) at this early time point (Fig. 3a, b).

At 6–12 months following combined revascularization poor bypass filling rates were stable compared to the first 6 months following surgery with 47% of STA-MCA and 39% of EDS hemispheres. The rate of moderate bypass filling increased among EDS to 57% of hemispheres with the rate in STA-MCA bypass remaining similar to the earlier time point at 45%. The rates of high bypass filling rose slightly in STA-MCA from 7 to 9% of hemispheres at this time point while EDS remained similar at 4% (versus 5% at 3–6 months) (Fig. 3a, b).

At 12–24 months following combined revascularization poor filling rates remained stable compared to the earlier time points among STA-MCA (49%) with EDS at 39% of hemispheres. An increase in the rate of high bypass filling could be seen in 14% of STA-MCA and EDS hemispheres. Moderate bypass filling remained similar in both groups at 51% (STA-MCA) and 47% (EDS) of hemispheres (Fig. 3a and b).

At time points longer than 24 months following combined revascularization, the rates of poor and moderate bypass filling remained stable for STA-MCA (poor: 42%, moderate: 49% of hemispheres) with lower rates of high bypass filling compared to earlier time points of 12–24 months (9% versus 14%). EDS showed a further increase in high bypass filling to 23% from 14% of hemispheres at 12–24 months. Accordingly, rates of poor bypass filling decreased by 6% and moderate filling by 3% (Fig. 3a, b).

Overall, when comparing STA-MCA function over all time points for each hemisphere individually until the latest available follow-up, we found that 3% of bypasses showed a regression of function with 52% remaining stable and 45% showing an increase of angiographic filling. In contrast, a higher proportion of EDS showed a regression of bypass filling over time (18%) with 34% remaining stable and 48% improving over time.

### Subgroup analysis

We further assessed the longitudinal changes among 14 hemispheres in 9 patients with complete sequential follow-up. In this small cohort, we found that at 3–6 months STA-MCA showed higher rates of poor filling versus EDS (67 vs. 47%), respectively (Fig. 3c, d). Over time, EDS evolved differently from the overall cohort, with a primary decrease in bypass filling, which was 100% poor from 12 to 24 months. However, moderate bypass filling increased to 29% at 24 months, reflecting a trend toward better function over time. The STA-MCA rate showed increased rates of higher filling at 6–12 months than at the earlier time point (33% vs. 0%, respectively), which then decreased over time. At time points longer than 24 months, high STA-MCA bypass filling was found in 6% of hemispheres. The proportion of poor STA-MCA bypass filling was high with 82% in this subgroup (Fig. 3c–d). Importantly, two of five bypass occlusion cases within 6 months following revascularization surgery were in this subgroup.

#### Development of EDS in MMS versus MMD

**Fig. 4:**
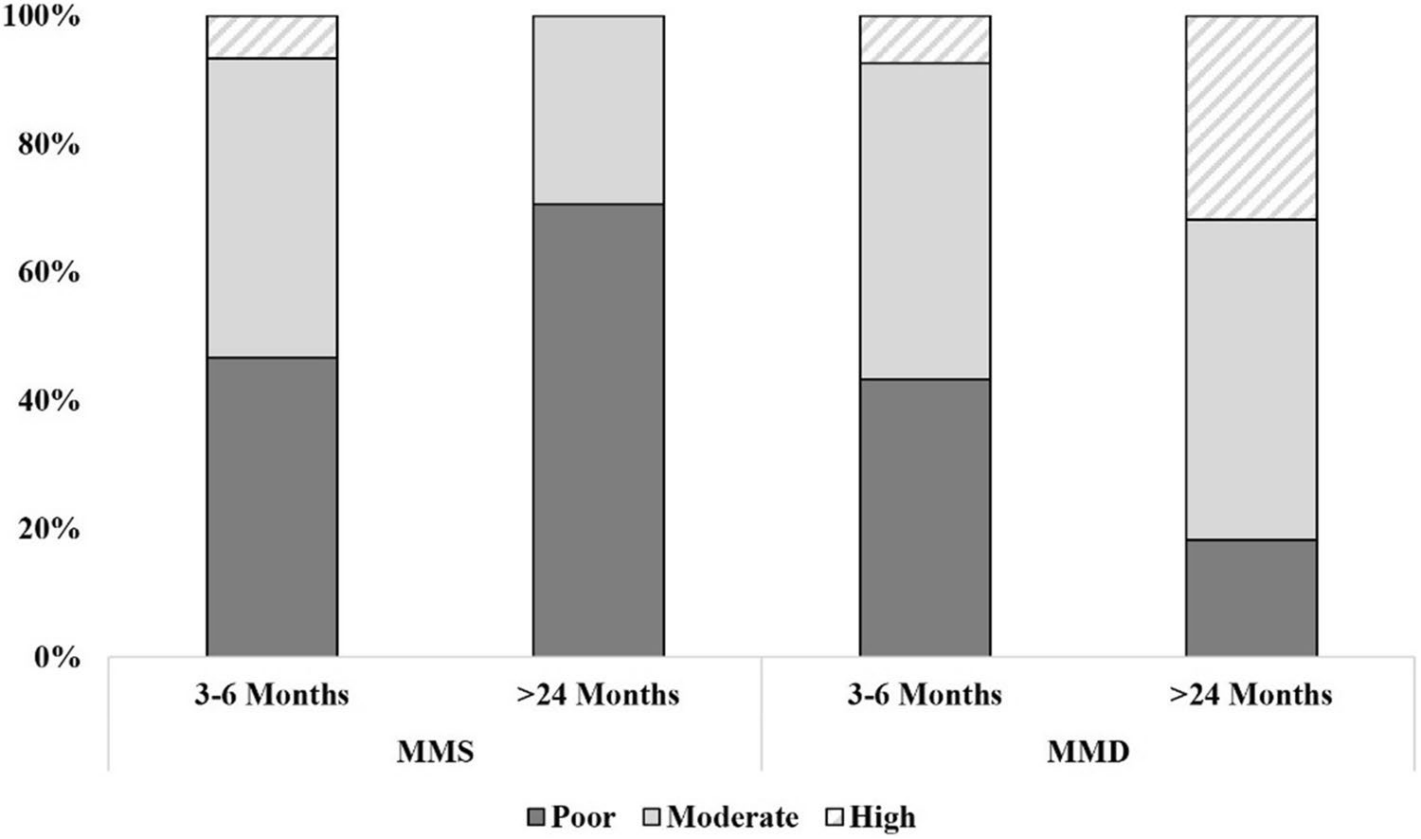
EDS Development in MMS versus MMD. EDS development in MMS (15 hemispheres at 3–6 months and 7 hemispheres at > 24 months) as well as MMD (124 hemispheres at 3–6 months and 7 hemispheres at >24 months)1). Percentages represent the number of hemispheres being scored as grade 1 (poor), 2 (moderate), or 3 (high) as a total of all hemispheres undergoing analysis for that particular time period. Analysis was performed at these time periods due to missing data in the MMS subgroup for the time periods 6–12 and 12–24 months. At 3–6 months 47% poor, 47% moderate, and 7% high filling in MMS patients with 43% poor, 49% moderate, and 7% high filling in MMD patients. At time points > 24 months 71% poor, 29% moderate, and 0% high filling in MMS with 18% poor, 50% moderate, and 32% high filling in MMD patients

Among the 13 MMS patients (25 hemispheres) in our cohort the rates of EDS high bypass filling at early time points (3–6 months) were the same as those in the remaining patients (*n* = 114 hemispheres) classified here as moyamoya disease (MMD) at 7% each, whereas MMD patients followed the trend of increased EDS bypass filling over time (32% at time points beyond 24 months) which was observed for the overall cohort, this development stagnated in patients with MMS (0% high bypass filling at time points above 24 months; Fig. 4).

#### Bypass occlusion and postoperative complications

Four patients (4.9%) experienced a postoperative complication following revascularization surgery. Of these, two were wound healing disorders and two were ischemic events related to surgery. The majority of patients undergoing bypass surgery had no bypass occlusion over the period of observation (90.1%). Five patients in our cohort experienced STA-MCA bypass occlusion of one hemisphere within 6 months following surgery, two cases of bypass occlusion occurred 3.5 years following surgery and two patients experienced bypass occlusion at 5.5 and 6.1 years. Of these, one case of neurological deterioration (recurrent TIAs) was reported resulting in repeat revascularization surgery using a frontal branch of the STA. This case of bypass occlusion occurred within 6 months following initial surgery and the development of EDS filling was rated as “poor.” In the remaining four hemispheres which demonstrated asymptomatic STA-MCA occlusion, angiographic filling of EDS was rated “moderate” or “high.” In these patients, no specific trigger for bypass occlusion was identified.

#### Predictors of EDS development

Finally, we examined the relationship between several demographic and angiographic factors and the development of EDS function over time. Using univariate cox regression analysis with good EDS outcome defined as a dependent variable, we found that at all time points only the degree of STA-MCA bypass filling was significantly associated with poor EDS bypass filling (Tables 2, 3, 4, and 5). Multivariate regression analysis performed on the variables preoperative deficit, history of TIA (3–6 months postoperatively), Berlin grade, STA-MCA grades, Suzuki stage, and age (6–12 months postoperatively) as well as preoperative cerebrovascular reserve capacity (CVRC) revealed no new significant predictors of EDS development, while STA-MCA bypass filling remained significant (Table 6).

**Table 2:** Model summary of regression analysis. Model summary of regression analysis on moderators analyzed with “moderate or high bypass filling” being defined as the dependent variable. *P* < 0.05. This table shows univariate regression analysis models

3–6 months					
	Hazard ratio	*P*	CI		
Berlin grade	− 0.165	0.471	− 0.222	-	0.104
Suzuki stage	0.262	0.266	− 0.068	-	0.241
Age	0.037	0.787	− 0.010	-	0.013
Preoperative deficit	0.239	0.115	− 0.067	-	0.602
History of stroke	− 0.031	0.822	− 0.331	-	0.264
History of TIA	0.225	0.143	− 0.098	-	0.660
History of hemorrhage	− 0.025	0.867	− 0.557	-	0.470
Leptomeningeal collaterals	0.057	0.670	− 0.493	-	0.761
Intra-extracranial collaterals	− 0.153	0.222	− 0.713	-	0.169
***STA-MCA bypass***	***0.334***	***0.016***	***0.062***	-	***0.566***
Moyamoya vessels decreased	0.015	0.91	− 0.482	-	0.540
Preoperative CVRC	0.768	0.315	0.315	-	1.871

**Table 3:** Model summary of regression analysis. Model summary of regression analysis on moderators analyzed with “moderate or high bypass filling” being defined as the dependent variable. *P* < 0.05. This table shows univariate regression analysis models

6–12 months					
	Hazard ratio	*P*	CI		
Berlin grade	0.422	0.061	− 0.009	-	0.370
Suzuki stage	− 0.334	0.143	− 0.286	-	0.043
Age	− 0.245	0.139	− 0.027	-	0.004
Preoperative deficit	− 0.180	0.253	− 0.577	-	0.157
History of stroke	0.073	0.633	− 0.273	-	0.442
History of TIA	− 0.079	0.634	− 0.584	-	0.361
History of hemorrhage	− 0.056	0.723	− 0.766	-	0.537
Leptomeningeal collaterals	− 0.220	0.186	− 0.614	-	0.124
Intra-extracranial collaterals	0.218	0.169	− 0.090	-	0.491
***STA-MCA bypass***	***0.491***	***0.004***	***0.150***	-	***0.726***
Moyamoya vessels decreased	− 0.007	0.964	− 0.260	-	0.559
Preoperative CVRC	1.042	0.930	0.419	-	2.593

**Table 4:** Model summary of regression analysis. Model summary of regression analysis on moderators analyzed with “moderate or high bypass filling” being defined as the dependent variable. *P* < 0.05. This table shows univariate regression analysis models

12–24 months					
	Hazard ratio	*P*	CI		
Berlin grade	− 0.034	0.922	− 1.371	-	0.906
Suzuki stage	− 0.120	0.743	− 1.740	-	2.069
Age	− 0.051	0.850	− 0.077	-	0.079
Preoperative deficit	− 0.024	0.904	− 1.939	-	1.410
History of stroke	0.021	0.913	− 1.986	-	1.853
History of TIA	− 0.079	0.768	− 3.955	-	4.241
History of hemorrhage	− 0.297	0.214	− 0.141	-	0.702
Leptomeningeal collaterals	− 0.249	0.318	− 0.619	-	0.838
Intra-extracranial collaterals	− 0.363	0.159	− 0.859	-	0.154
***STA-MCA bypass***	***0.481***	***0.006***	***0.214***	-	***0.969***
Moyamoya vessels decreased	0.075	0.753	− 2.543	-	3.215
Preoperative CVRC	1.768	0.534	0.294	-	10.634

**Table 5:** Model summary of regression analysis. Model summary of regression analysis on moderators analyzed with “moderate or high bypass filling” being defined as the dependent variable. *P* < 0.05. This table shows univariate regression analysis models

> 24 months					
	Hazard ratio	*P*		CI	
Berlin grade	− 0.219	0.455	− 0.367	-	0.172
Suzuki stage	0.075	0.817	− 0.222	-	0.277
Age	0.005	0.981	− 0.020	-	0.020
Preoperative deficit	0.135	0.422	− 0.251	-	0.571
History of stroke	0.074	0.675	− 0.359	-	0.540
History of TIA	− 0.058	0.771	− 0.814	-	0.614
History of hemorrhage	− 0.063	0.740	− 1.079	-	0.782
Leptomeningeal collaterals	− 0.155	0.442	− 0.843	-	0.386
Intra-extracranial collaterals	− 0.339	0.168	− 0.989	-	0.187
***STA-MCA bypass***	***0.636***	***0.002***	***0.336***	-	***1.227***
Moyamoya vessels decreased	− 0.095	0.631	− 0.741	-	0.462
Preoperative CVRC	1.136	0.899	0.159	-	8.099

**Table 6:** Displays results of multivariate regression analysis using variables from the univariate analysis which gave a *p*-value of < 0.15

	Hazard ratio	*P*	CI		
3–6 months					
Preoperative deficit	− 0.115	0.477	− 0.511	-	0.243
History of TIA	0.119	0.396	− 0.204	-	0.510
* STA-MCA bypass*	***0.461***	***0.003***	***0.151***	***-***	***0.673***
6–12 months					
Berlin grade	0.439	0.272	− 0.218	-	0.662
Suzuki stage	− 0.833	0.201	− 0.990	-	0.250
Age	− 0.193	0.204	− 0.023	-	0.005
* STA-MCA bypass*	***0.469***	***0.022***	***0.096***	***-***	***1.122***
12–24 months					
-					
> 24 months					
-					

Tables 2, 3, 4, and 5 show univariate regression analysis models. Table 6 displays results of multivariate regression analysis using variables from the univariate analysis which gave a *p*-value of < 0.15. No variables meeting this criteria were available in the time periods 12–24 months and > 24 months; therefore, no results are depicted for these times. STA-MCA bypass development was classified as “poor” (no filling of the MCA territory), “moderate” (antegrade filing of one or two MCA branches), or “high” (complete filling of the MCA territory) [9]. EDS development was classified as “poor” (no filling), moderate (opacification of cortical vessels), or “high” (filling of the MCA territory) [12]. Good leptomeningeal collateralization preoperatively was determined based on the presence of anastomoses in the watershed zones of the posterior cerebral artery to the anterior and/or to the middle cerebral artery and poor collateralization was determined in absence of the above anastomoses [18]. Intra-extracranial collateralization was examined based on the preoperative presence and degree (none, moderate, extensive) of transdural collaterals arising from the middle meningeal artery [19, 20]. Development of moyamoya vessels was classified as either absent, faint and localized or abundant [12].

## Discussion

Although the use of combined bypass strategies in the treatment of both hemorrhagic and ischemic MMA is supported by studies demonstrating positive angiographic, hemodynamic, and clinical results [6, 9, 21–24], the use of EDS as a method of indirect revascularization combined with STA-MCA bypass has remained uncharacterized despite its simplicity and ability to perform in a minimally invasive fashion.

Several studies on of EDAS [13, 25] and EMS [9, 26] alone or in combination with STA-MCA bypass have demonstrated increased vessel growth on angiography. However, specific evaluation of EDS has rarely been performed. One study in hemorrhagic-onset MMA patients has analyzed angiographic outcome in a subset of patients (24 hemispheres) receiving combined revascularization using EDS compared to EDAS (30 hemispheres) [27]. In this series, angiographic development as measured by the Matsushima revascularization level showed no statistically significant difference between the EDS and EDAS groups. As EDS requires less surgical exposure than the abovementioned techniques, these results suggest that its use may allow for comparable angiographic outcome with less invasiveness.

Despite initial evidence that EDS may provide good angiographic outcome compared to more invasive techniques, our study is to the best of our knowledge, the first to characterize the outcome of EDS in combination with direct bypass in a large cohort of hemorrhagic and ischemic onset MMA. In particular, we describe the use of a MIS-EDS via a small craniotomy approximately 3 cm in diameter which even further reduces the invasiveness of the overall procedure.

Our cohort consisting of 139 hemispheres with 89% ischemic onset and median age of 44 years was representative in comparison to other Caucasian MMA series [28, 29]

Here, we did indeed observe that STA-MCA direct bypass showed better angiographic filling at an earlier time point than EDS. STA-MCA function was found to be highest at earlier time points following initial revascularization and then stagnates at this level after 12 months following surgery. At time points after 12 months following combined revascularization surgery, EDS bypass filling began to improve whereas STA-MCA stagnated. Although performed in a smaller subgroup, these trends could also be observed in patients for which angiographic data was available across all four time points. Our results differ slightly from previous angiographic analysis of an Asian cohort consisting of 25 hemispheres receiving combined bypass surgery using EDAS alone and follow-up at 6 and 12 months [30]. In these patients, maximal neoangiogenesis from indirect bypass was found already at 6 months. This difference to our results may have several reasons, including the use of indirect bypass alone, smaller sample size, and follow-up conducted only up to 12 months following surgery in the Asian cohort. A previous analysis from our institution on EMS bypass development alone and in combination with STA-MCA at 6 and 12 months following revascularization demonstrated similar dynamics of bypass function when compared to our results for EDS [9]. In particular, EDS and EMS both show an increase in the proportion of high bypass filling at later time periods (12 versus 6 months), whereas STA-MCA bypass function shows good function earlier. These findings support the recommendation of aiming for direct revascularization whenever possible in order to provide immediate support for insufficient reserve capacity [7].

In our analysis of the subset of 14 hemispheres for which all observation periods were available, we first confirmed the main trend observed within the overall cohort in which EDS developed over a longer period. Compared to the overall cohort, both EDS and STA-MCA in the subgroup had higher proportions of poor bypass filling over nearly all time points. We hypothesize that this is due to the selection bias in the comparatively small sample size of the subgroup (14 hemispheres) versus the overall cohort (139 hemispheres). In addition, of the five patients in the overall cohort who experienced bypass occlusion, two are included in this subgroup of 14 hemispheres. In these patients occlusion occurred at time points longer than 2 years and patients were neurologically asymptomatic despite STA-MCA bypass filling being poor at time points up until occlusion. Continuing follow-up of all treated patients will provide further insights over time.

To the best of our knowledge, the role of EDS has not yet been examined within the context of MMS versus MMD or uMMD. In our initial analysis of 25 hemispheres from patients with MMS, we found that EDS bypass development did not increase over time as was found in patients with MMD or uMMD. As our analysis was only performed on a small subset of patients within our cohort, further studies would be warranted to further explore and validate these initial results, which may have implications for individualized revascularization strategies according to disease variants.

In our study, Cox regression analysis showed that the grade of STA-MCA bypass development was significantly associated with EDS bypass development, with better STA-MCA bypass filling predicting poor EDS angiographic filling. Functional adaptation of direct anastomoses is hypothesized to rely mainly on pressure gradients from donor to recipient arteries, whereas development of indirect anastomoses involves several precluding steps of angiogenesis and arteriogenesis [26]. Accordingly, angiographic evidence of bypass perfusion and development of vascular networks may be expected to arise sooner in direct bypasses than indirect [7], therefore possibly mitigating the angiogenic signals which would otherwise promote EDS development in compensation for insufficient direct bypass function. This question of predictive factors for bypass neoangiogenesis has been addressed in previous studies which have focused on hemorrhagic-onset MMA. First, a multicenteric analysis of 231 hemispheres from patients undergoing various indirect revascularization procedures found that hemorrhagic disease onset positively predicts poor neovascularization and that abundant moyamoya vessels were positively associated with good postoperative neovascularization resulting from indirect bypass [12]. We observed no statistically significant relationship between angiographic grading Suzuki or Berlin grading and the development of EDS, which is concurrent with a recent report on the development of EDAMS in an Indian population [31]. Secondly, neoangiogenesis and good bypass function have also been previously described to occur more frequently in pediatric than adult patients [18]. In contrast to this study, our analysis revealed no statistically significant relationship between age and degree of indirect bypass development which may be due to the fact that our pediatric patients were teenagers closer to 18 years of age rather than younger children. Children also made up only a very small part of our total collective with 4/81 patients being under the age of 18. A further study on a smaller cohort consisting of 64 operated hemispheres found that only the location of initial hemorrhage in the anterior territory was predictive of good postoperative collateral formation [32]. The use of different grading systems of postoperative neoangiogenesis between these two analyses may have contributed to their varying conclusions. As our study included only a minority of hemorrhagic-onset cases a direct comparison of results is challenging.

Although angiographic bypass performance is not a guaranteed indicator of clinical stability good angiographic revascularization from indirect bypass (EDAS) has been shown to positively associate with lower incidence of hemorrhage and improvement of neurological symptoms in patients with hemorrhagic-onset MMD [12] In patients undergoing direct STA-MCA revascularization there is also evidence for reduced secondary stroke in cases with good angiographic revascularization [33]. In our cohort, angiographic direct bypass occlusion was found in a total of eight hemispheres, one of which required repeat revascularization using a frontal STA branch due to persisting neurological symptoms. This patient also showed poor EDS development. In the remaining seven hemispheres, there was STA-MCA occlusion with good or moderate angiographic filling of EDS. These observations support the hypothesis that combined revascularization with EDS may provide a second line of protection against ischemic insult in case of direct bypass failure similar to EMS [9]. As the EDS procedure described in our study does not require any additional invasiveness during the direct revascularization surgery, it can easily be transferred to the clinical routine. The postoperative complication rate was lower than that of EMS reported previously [9], which could be attributed to the minimally invasive approach via a mini-craniotomy. Nevertheless, due to the relatively small surface of the EDS through the mini-craniotomy, the hemodynamic functionality in addition to angiographic development should be further assessed in the future.

Limitations of the current study include its retrospective design with the majority of adult patients which may skew interpretation of factors affecting bypass development in terms of children versus adults. Furthermore, as patients from a wide range of countries across Europe are treated in our institute, the availability for regular on-site follow-up is somewhat limited so that data at all four time point was only available for a subgroup of patients. As this analysis focused on angiographic outcome, data on the development of cerebrovascular reserve capacity over time was not included as this is not regularly performed following surgery in asymptomatic patients, also due to the possible side effects of repetitive acetazolamide and radionuclide application. Thus, the functional contribution of EDS in addition to STA-MCA remains unquantified. Although such analysis may provide more insight into the functional consequences of good or poor bypass development over time, the clinical outcome of patients remains the most important factor in daily neurosurgical practice. As EDS is a procedure which is not performed alone, but only in combination with direct bypass we do not have control group in which only EDS can be compared to. In this regard, a further limitation of our study is that we have no patients which were treated with EDS alone in order to elucidate the development and clinical effect of EDS without the contribution of STA-MCA vascularization. This however lies in the nature of the procedure which is not a stand-alone technique. Nonetheless, EDS merits evaluation as it continues to be implemented in the clinical routine, and as our study shows can be performed in a minimally invasive fashion with angiographic benefit.

## Conclusion

To the best of our knowledge, this study represents the largest cohort in which long-term angiographic characterization of combined revascularization with EDS and STA-MCA direct bypass development has been performed. While there are currently no randomized control trials regarding the benefit of different bypass strategies, in our experience EDS is a safe technique of indirect revascularization for MMA and can be used in a standardized fashion through a small craniotomy for both pediatric and adult patients. Despite the use of EDS in clinical practice, until now it has remained unclear whether there is an angiographic benefit. Our results demonstrate that EDS may offer a reasonable option to perform combined revascularization in patients with moyamoya disease and serves as a second line of protection with angiographic evidence of vascularization resulting from EDS. Prospective comparative studies are warranted to address the efficacy of this combined technique in comparison to direct bypass only.

## Data Availability

Anonymized data is available upon reasonable written request.
